# Interaction of the growth and tumour suppressor NORE1A with microtubules is not required for its growth-suppressive function

**DOI:** 10.1186/1756-0500-1-13

**Published:** 2008-05-15

**Authors:** Anna Moshnikova, Sergey Kuznetsov, Andrei V Khokhlatchev

**Affiliations:** 1 Department of Pathology, University of Virginia, PO Box 800904, Charlottesville, VA 22908-0904, USA

## Abstract

**Background:**

The NORE1 protein was identified in a yeast two-hybrid screen as a Ras effector that binds Ras protein in a GTP-dependent manner. NORE1A is a growth and tumour suppressor that is inactivated in a variety of cancers. In transformed human cells, both full-length NORE1A protein and its effector domain alone (amino acids 191–363) are localized to microtubules and centrosomes. However, the mechanism by which NORE1A associates with these cytoskeletal elements is not known; furthermore, whether centrosomally-associated or microtubule-associated NORE1A suppresses tumour cell growth has not been yet established.

**Findings:**

We have shown that purified NORE1A fails to bind to microtubules *in vitro *suggesting that other protein(s) mediate NORE1A-microtubule association. Using mass-spectrometry, we identified the Microtubule-Associated Protein 1B (MAP1B) and its homologue C19ORF5 as NORE1A interaction partners. Suppression of C19ORF5 expression by RNA interference (RNAi) and immunodepletion of C19ORF5 protein from cell extracts showed that binding of NORE1A to microtubules is not dependent on C19ORF5. Conversely, RNAi suppression of MAP1B revealed that MAP1B is required for association of NORE1A with microtubules. RNAi-mediated depletion of C19ORF5 or MAP1B did not prevent centrosomal localization of NORE1A. Moreover, the depletion of C19ORF5 or MAP1B did not prevent NORE1A's ability to suppress tumour cell growth.

**Conclusion:**

The interaction of NORE1A with microtubules is mediated by MAP1B, but not C19ORF5 protein. Interaction of NORE1A with centrosomes is not dependent on C19ORF5 or MAP1B, and appears to involve a different mechanism independent of binding to microtubules. The NORE1A microtubular localization is not required for growth suppression.

## Findings

### NORE1A interacts with C19ORF5 and MAP1B proteins

**Figure 1:**
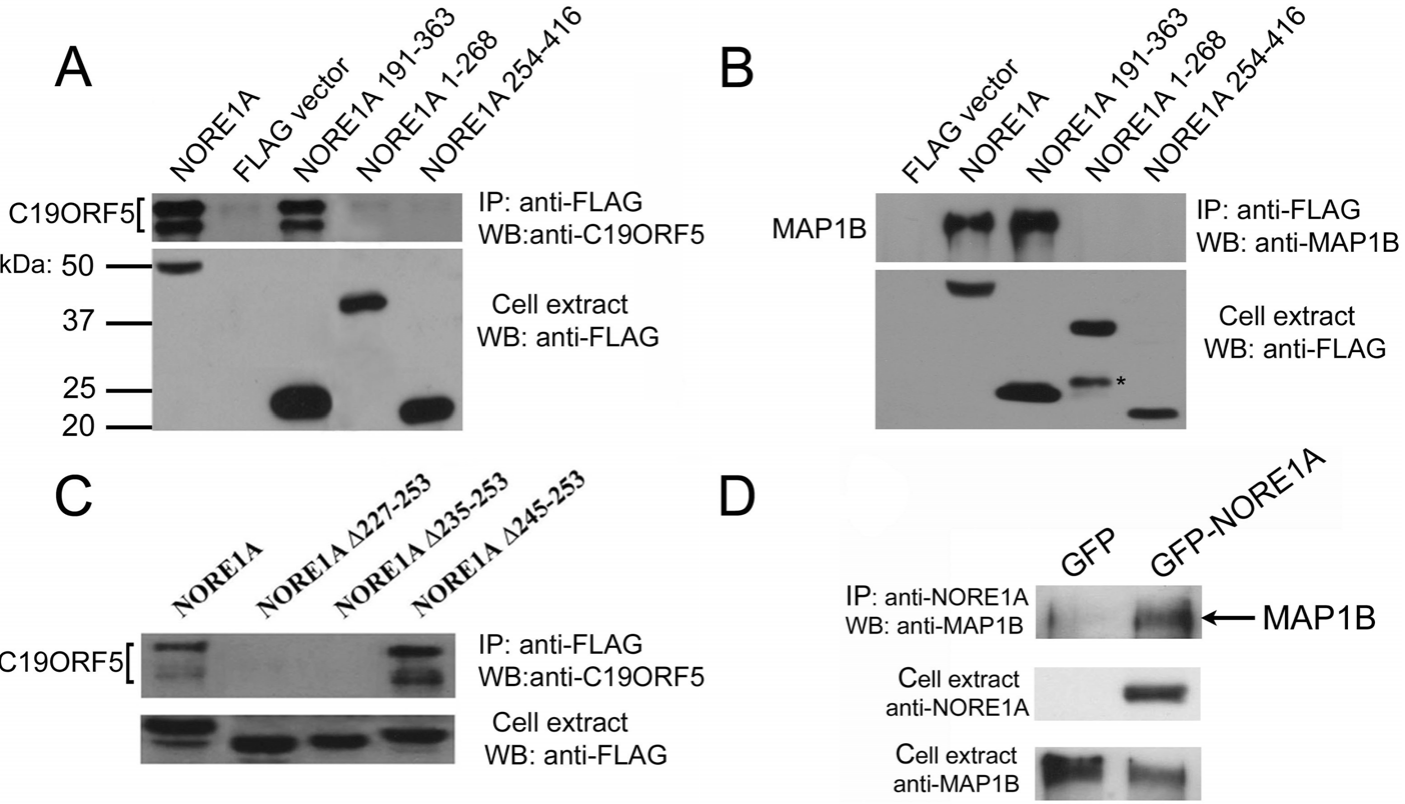
**Interaction of C19ORF5 and MAP1B proteins with wild type and mutant NORE1A**. **A-C**: HEK293 cells were transfected with FLAG-tagged wild type NORE1A, fragments of NORE1A, or deletion mutants as indicated. Cell lysates were precipitated for FLAG and the precipitate was probed with antibody against C19ORF5 and MAP1B (upper panels). Lower panels show expression of FLAG-tagged proteins in cell extracts. Asterisk denotes NORE1A 1–268 degradation product. **D**: A549 cells were infected with retrovirus coding for hrGFP-tagged wild type NORE1A or hrGFP alone as indicated. Cell extracts were precipitated for NORE1A and the precipitate was probed with antibody against MAP1B (upper panel). Lower panels show expression of NORE1A and MAP1B in cell extracts.

We have previously shown that NORE1A associates with microtubules and that this association is most likely indirect since purified NORE1A cannot efficiently bind to microtubules assembled from pure tubulin [1]. To identify proteins that mediate association of NORE1A with microtubules, we expressed FLAG-tagged full-length NORE1A or effector domain (amino acids 191–363) in HEK293 cells and examined NORE1A-bound proteins by mass spectrometry. Among the most abundant NORE1A protein partners, we identified two distinct microtubule-binding proteins: C19ORF5 and MAP1B. The interaction between ectopically expressed NORE1A and endogenous C19ORF5 and MAP1B proteins was confirmed by coimmunoprecipitation (Figs. 1A–B and 1D).

### C19ORF5 interacts with NORE1A but does not mediate its binding to microtubules

Both full-length NORE1A and its effector domain alone are capable of interacting with endogenous C19ORF5 protein in HEK293 cells (Fig. 1A). The N-terminal portion (amino acids 1–268) and the C-terminal portion (amino acids 250–416) of NORE1A failed to interact with C19ORF5 (Fig. 1A), suggesting that the intact effector domain, amino acids 191–363, mediates NORE1A-C19ORF5 interaction.

To map the NORE1A-C19ORF5 interaction interface more precisely, we made several small deletions in the NORE1A effector domain and tested these proteins for interaction with C19ORF5. Figure 1C shows that the deletion of amino acids 227–253 completely abolished NORE1A-C19ORF5 interaction. The smaller deletion mutant, NORE1A Δ236–253, also failed to interact with C19ORF5; however, the deletion mutant NORE1A Δ246–253 retained binding activity. Thus, the region 227–246 within the NORE1A effector domain is responsible for interaction with the C19ORF5 protein.

**Figure 2:**
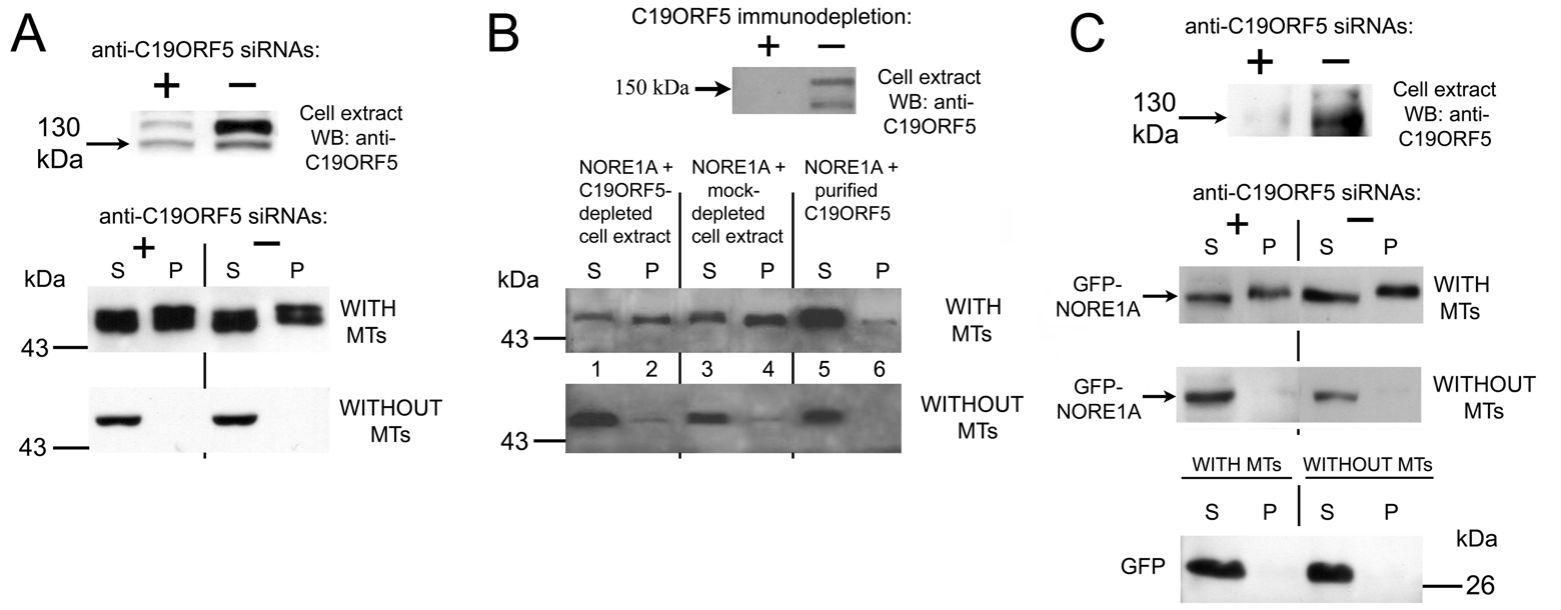
**The C19ORF5 protein does not mediate NORE1A binding to microtubules *in vitro***. **A**: C19ORF5 protein was depleted by RNA interference in HEK293 cells expressing HA-tagged NORE1A. Equal amounts of cell extracts were probed for C19ORF5 (upper panel) or used to examine the ability of NORE1A to bind to microtubules using the microtubule cosedimentation *in vitro *assay. **B**: HEK293 cell extract was immunodepleted of the C19ORF5 protein by incubation with 4G1 antibody followed by Protein A/G plus agarose, or mock-depleted. Equal amounts of C19ORF5-depleted and mock-depleted extracts were probed for C19ORF5 (upper panel). The ability of purified FLAG-NORE1A to interact with microtubules after preincubation with C19ORF5-immunodepleted extract (lanes 1–2), mock-immunodepleted extract (lanes 3–4), or purified FLAG-C19ORF5 (lanes 5–6) was examined as described in the Methods. **C**. C19ORF5 protein was depleted by RNA interference in A549 cells expressing GFP-tagged NORE1A. Equal amounts of cell extracts were probed for C19ORF5 (upper panel) or used to examine the ability of NORE1A to bind to microtubules as described in Figure 1 (two middle panels). Lower panel, the ability of GFP moiety, expressed alone in A549 cells, to bind to microtubules was examined.

To determine whether C19ORF5 protein is required for the interaction of NORE1A with microtubules, we first depleted endogenous C19ORF5 protein using RNAi and examined the interaction of NORE1A with microtubules in an *in vitro *cosedimentation assay. Despite significant RNAi-mediated depletion of the endogenous C19ORF5 protein from HEK293 cells expressing NORE1A, the ability of NORE1A to interact with microtubules was not diminished (Fig. 2A). To completely remove C19ORF5, we immunodepleted the HEK293 cell extract with anti-C19ORF5 antibody. Purified NORE1A was then added to immunodepleted or mock-depleted cell extract and the ability of NORE1A to interact with microtubules was examined. Figure 2B shows that, despite efficient C19ORF5 immunodepletion (upper panel), the depleted cell extract was as capable of mediating NORE1A interaction with microtubules as mock-depleted extract (middle panel, lanes 1–4). In addition, purified C19ORF5 protein could not induce interaction of purified NORE1A with microtubules (Figure 2B, middle panel, lanes 5 and 6), although the purified C19ORF5 itself interacted strongly with microtubules (data not shown). The depletion of C19ORF5 protein using RNA interference in A549 cells expressing GFP-NORE1A also did not diminish the ability of GFP-NORE1A to interact with microtubules *in vitro *(Fig. 2C).

**Figure 3:**
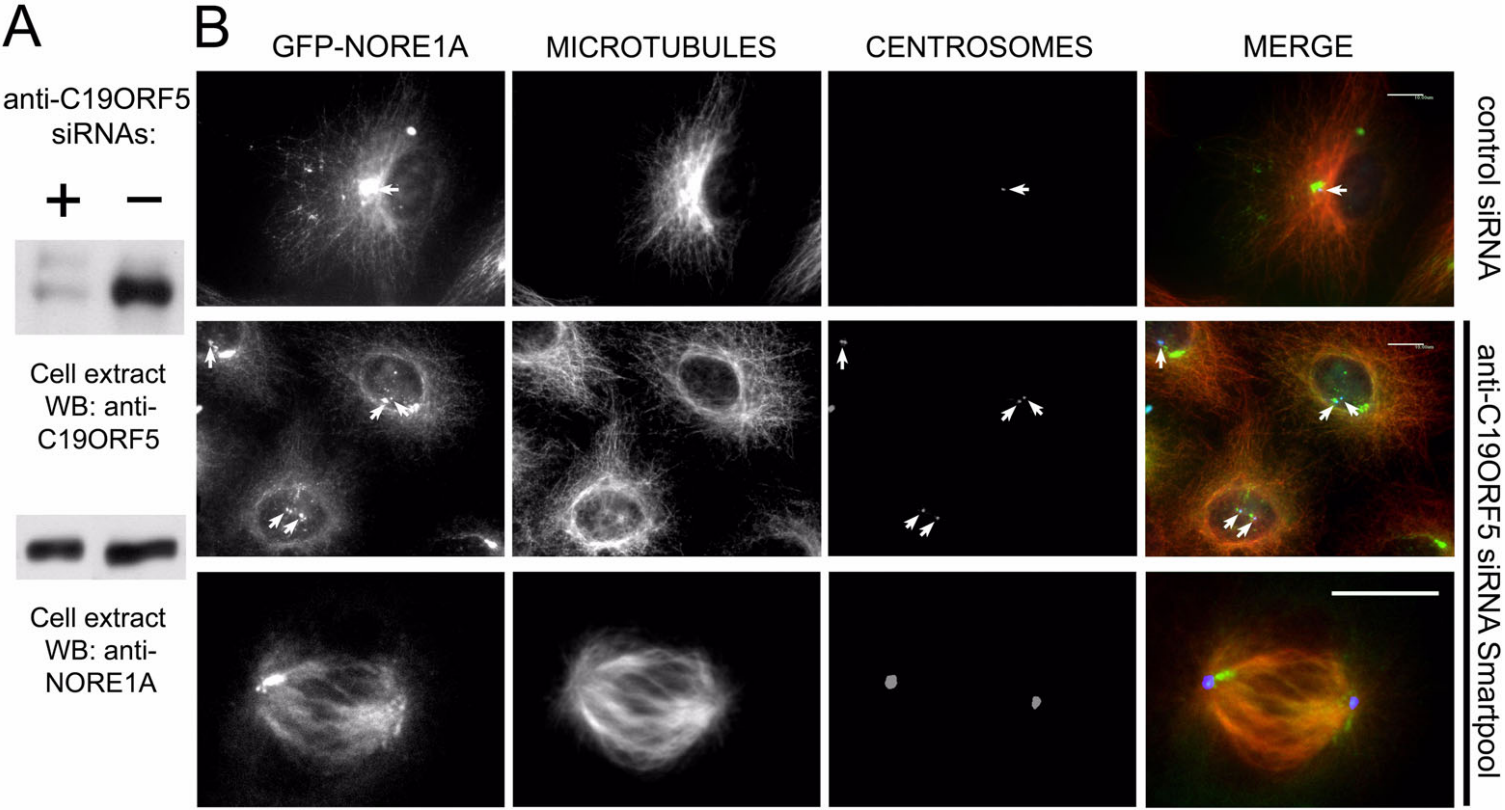
**The C19ORF5 protein does not mediate NORE1A binding to microtubules in living cells**. **A, B**: A549 cells expressing GFP-NORE1A were transfected with anti-C19ORF5 siRNA pool or control siRNA as described in the Methods. An aliquot of cells was used to examine efficiency of C19ORF5 depletion (**A**, upper panel) and NORE1A expression level (**A**, lower panel) while other cells growing on coverslip were fixed, processed for immunofluorescence and imaged as described in Methods (**B**). In **B**, each panel shows NORE1A, microtubules (stained with α-tubulin), centrosomes (stained with pericentrin) and a superimposed image. Arrows indicate centrosomes. Bar, 10 μm.

We have shown previously that full-length NORE1A or its effector domain, tagged with N terminal GFP, localizes to microtubules and centrosomes in living A549 cells [1]. We used this cell system combined with RNAi to examine the role of C19ORF5 in NORE1A intracellular distribution. Figure 3A upper panel shows that the C19ORF5 protein was efficiently depleted from A549 cells; importantly, this depletion has no effect on the GFP-NORE1A abundance (Fig. 3A, lower panel). However, the GFP-NORE1A associated with microtubules (visualized by α-tubulin staining) as efficiently in C19ORF5-depleted cells as in control siRNA-depleted cells (Figure 3B). The colocalization was observed in both interphase (Fig. 3B, middle row) and mitotic (Fig. 3B, bottom row) cells. These results indicate that C19ORF5 is not required for targeting of NORE1A to microtubules *in vivo*.

### MAP1B interacts with NORE1A and is required for its binding to microtubules

MAP1B is a large (ca. 2500 amino acids) microtubule-associated protein consisting of several domains. Figures 1B and 1D show that NORE1A and the effector domain can interact with endogenous MAP1B protein in HEK293 and A549 cells, respectively. Furthermore, interaction required the intact NORE1A effector domain (amino acids 191–363) since neither the N-terminal portion (aa 1–268) or the C-terminal portion (aa 250–416) of NORE1A could interact with MAP1B (Fig. 1B).

**Figure 4:**
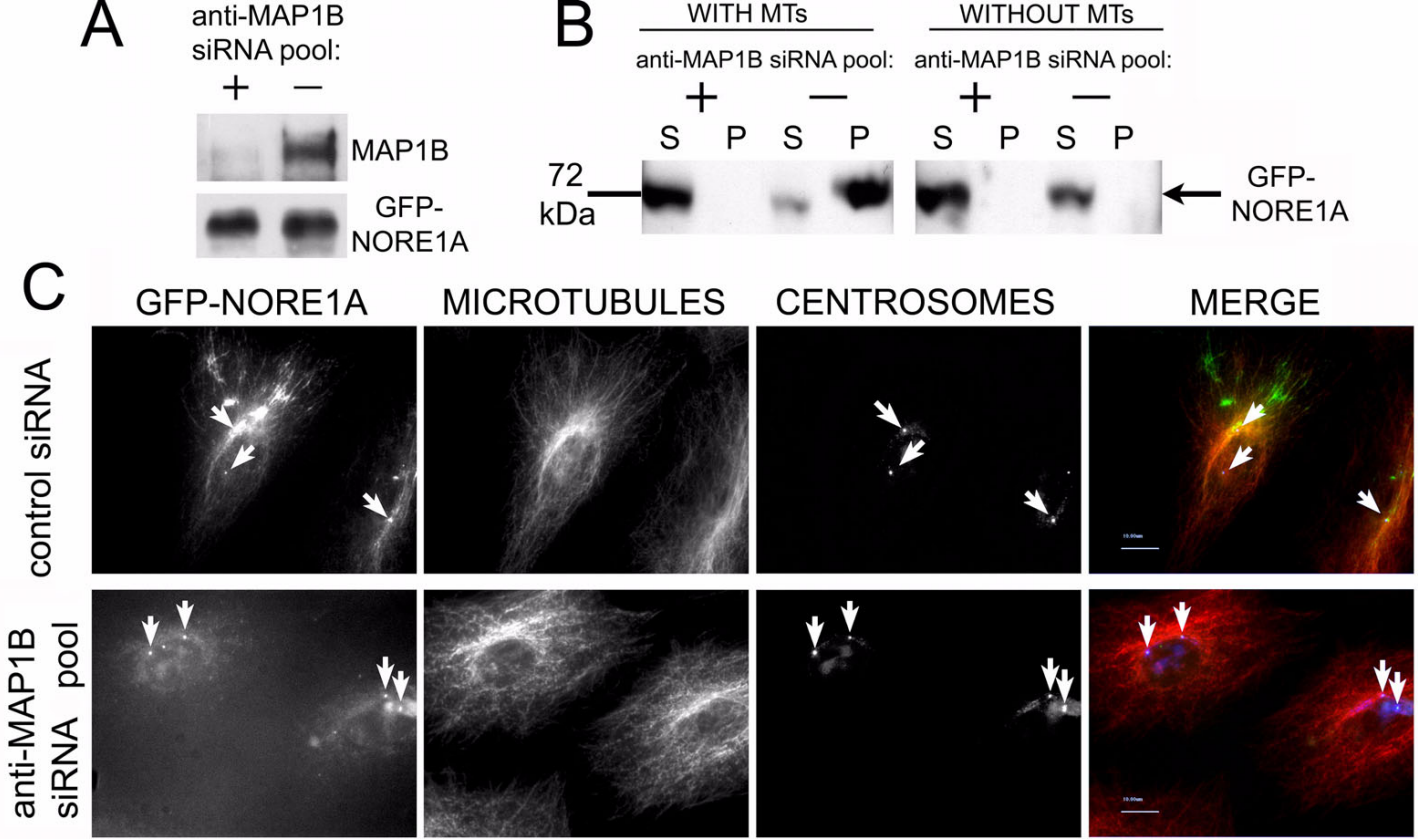
**The MAP1B protein is required for NORE1A binding to microtubules**. **A, B**: A549 cells were co-transfected with RNA oligos labelled with the Cy3 dye and anti-MAP1B siRNAs or control siRNA. After one day cells were infected with retrovirus encoding GFP-NORE1A. Two days later, cells were sorted using GFP and Cy3 markers, allowed to recover overnight, lysed and equal amounts of cell extract were probed for expression of MAP1B protein (**A**, upper panel) and GFP-NORE1A expression level (**A**, lower panel). Lysates of GFP and Cy3- positive cells were examined for the ability of GFP-NORE1A to interact with microtubules as described in Figure 1 **(B)**. **C**: A549 cells expressing GFP-NORE1A were transfected with anti-MAP1B siRNA pool or control siRNA. Cells were fixed, processed for immunofluorescence, and imaged as described in Methods. Each panel shows NORE1A, microtubules (stained with α-tubulin), centrosomes (stained with pericentrin) and a superimposed image. Arrows indicate centrosomes. Bar, 10 μm.

To determine whether MAP1B protein mediates the interaction of NORE1A with microtubules, we depleted endogenous MAP1B from A549 cells by RNAi and examined the ability of NORE1A or its effector domain to bind to microtubules *in vitro*. Figure 4A shows that endogenous MAP1B expression was virtually eliminated by RNAi in A549 cells and Figure 4B shows that NORE1A was unable to interact with microtubules in the absence of the MAP1B protein.

To determine whether MAP1B protein is required for targeting of NORE1A to microtubules in living cells, we examined the distribution of GFP-tagged NORE1A or its effector domain in A549 cells in which MAP1B has been depleted by RNAi. Depletion of MAP1B resulted in loss of microtubule localization of full-length NORE1A (Figure 4C) and its effector domain, aa 191–363 (See additional file 1: Supplemental Figure 2). Probing of A549 cell lysates where MAP1B has been depleted for NORE1A indicated that the NORE1A level was not changed, ruling out the effect of MAP1B on NORE1A stability (Fig. 4A, lower panel). Thus, our results indicate that the MAP1B protein is essential for interaction of NORE1A with microtubules *in vitro *and *in vivo*.

The reason why binding of NORE1A to microtubules is mediated by MAP1B, but not by C19ORF5 is not clear at the present time. For example, it is possible that the C19ORF5 protein mediates NORE1A interaction with microtubules under specific conditions or in specific cell types not tested here.

### The effect of MAP1B or C19ORF5 on centrosomal localization of NORE1A

We showed previously that NORE1A is localized to centrosomes in addition to microtubules [1]. This localization was observed for endogenous NORE1A, and for ectopically expressed NORE1A and its effector domain. Song and co-workers showed that the C19ORF5 protein was present on centrosomes in interphase and mitotic HeLa cells [2], while a recent report from the Latif group described centrosomal localization for C19ORF5 only in mitotic cells [3]. The MAP1B protein was also detected on centrosomes in interphase and mitotic cells [4,5]. We tested whether C19ORF5 or MAP1B play a role in the centrosomal localization of NORE1A.

In A549 cells into which NORE1A was introduced by retroviral-mediated gene transfer, depletion of the C19ORF5 protein did not result in dissociation of NORE1A from centrosomes in interphase (Fig. 3B, middle row) or mitotic (Fig. 3B, bottom row) cells. Depletion of MAP1B protein induced dissociation of full-length NORE1A (Fig. 4C, arrows) and its effector domain (See additional file 1: Supplemental Figure 3) from microtubules, but not from centrosomes. Thus, centrosomal localization of NORE1A is independent of its microtubular localization and does not require C19ORF5 or MAP1B proteins.

In contrast to NORE1A, the centrosomal localization of the closely related tumour suppressor RASSF1A was found to be dependent on the C19ORF5 protein [2], possibly reflecting differences in the regulation or mechanisms of action of NORE1A and RASSF1A. Alternatively, this difference could be caused by the use of different cell systems: HeLa cells in ref. [2], compared with A549 cells in this study.

### MAP1B or C19ORF5 are not required for NORE1A to suppress tumour cell growth

**Figure 5:**
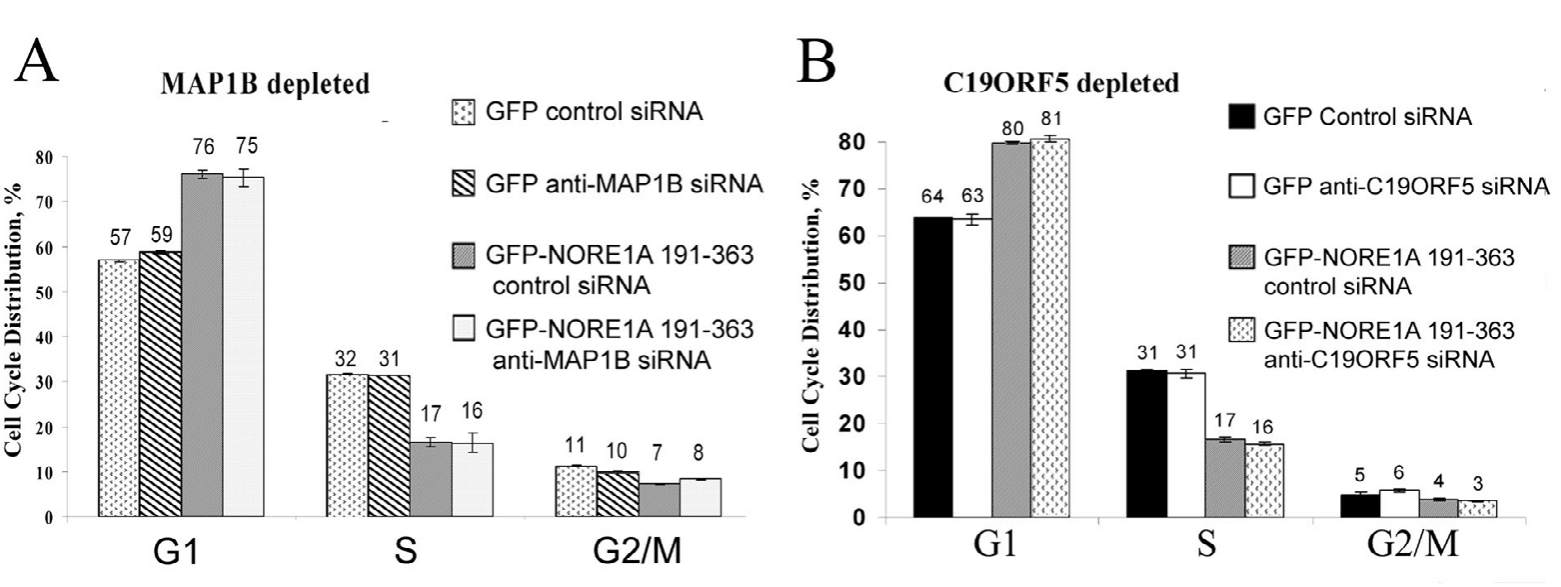
**C19ORF5 or MAP1B depletion does not interfere with NORE1A-induced growth suppression**. A549 cells were transfected with anti-MAP1B siRNA pool (**A**) or control siRNA, or anti-C19ORF5 siRNA pool or control siRNA (**B**) then infected with retrovirus expressing GFP or GFP-NORE1A effector domain (aa 191–363). Two days later, cells were fixed, processed for flow cytometry and analyzed as described in Methods. The percentage of GFP-positive cells in each phase of the cell cycle +/- mean is shown.

In an earlier study, we showed that the NORE1A mutant that was incapable to localize to both centrosomes and microtubules was deficient in growth suppression [1]. However, the issue whether microtubular localization or centrosomal localization of NORE1A is required for suppression of tumour cell growth remained unresolved. Here, we took advantage of the finding that full-length NORE1A and its effector domain dissociate from microtubules but not from centrosomes upon the MAP1B depletion. In A549 cells into which NORE1A effector domain was introduced by retroviral-mediated gene transfer, depletion of the MAP1B protein did not interfere with NORE1A's ability to suppress growth (Fig. 5A). Likewise, the depletion of the C19ORF5 protein (Fig. 5B) and the combined depletion of MAP1B and C19ORF5 (data not shown) have no effect on NORE1A's induced growth suppression.

Our previous work identified endogenous NORE1A as centrosomal protein in nontransformed human lung cells and as a centrosomal and microtubular protein in transformed lung cells [1]. However, this study shows that the microtubular NORE1A association is not required for its growth-suppressive activities. This raise the possibility that it is centrosomal NORE1A that is important for growth suppression. Further studies are required to determine the mechanism of NORE1A centrosomal localization and the importance of its targeting to centrosomes for suppression of tumour cell growth.

#### Reagents

Pools of small-interfering RNA duplexes, cat.# L-016881-00 against C19ORF5 and cat.# L-010348-00 against MAP1B were from Dharmacon, Inc. (Lafayette, CO). The Alexa Fluor 488-labelled AllStars Negative control siRNA, cat # 1027284, were from Qiagen (Valencia, CA). RNA duplex labelled with CY3 dye, cat # 4621, were from Ambion (Austin, TX). The anti-C19ORF5 4G1 antibody were from A&G Pharmaceutical, Inc. (Columbia, MD). The monoclonal anti-NORE1A antibody 10F10 was described in ref [1]. Anti-pericentrin antibody, cat.# ab-4448, were from Abcam (Cambridge, MA), the anti-α-tubulin DM-1-A and anti-γ-tubulin GTU-88 antibodies were from Sigma (St Louis, MO), and the anti-MAP1B antibody, cat # 612679 was from BD Biosciences. Secondary antibody conjugates were from Jackson ImmunoResearch (West Grove, PA). All other reagents were previously described by Moshnikova et al. [1].

#### Plasmids

Plasmids encoding NORE1A were described previously [1]. The human C19ORF5 protein (IMAGE clone; ID 5925583) was purchased from ATCC (Manassas, VA) and subcloned into pCMV5 vector with the N-terminal FLAG tag. This and all other constructs were prepared using standard molecular biology techniques [6] and verified by sequencing.

#### Cell Lines, Transfection and Retrovirus Production

Cultivation of A549 human lung adenocarcinoma and HEK293 human embryonic kidney cells (ATCC), HEK293 cells transfection and retroviral supernatants production was described previously [1,7]. The siRNA duplexes were transfected using DharmaFECT1 reagent (Dharmacon) according to the manufacturer's instructions.

#### Immunodepletion, Immunoprecipitation and Western Blot Analysis

HEK293 cells were lysed in buffer A (30 mM HEPES, pH 7.4, 20 mM KCl, 1mM NaVO_4_, 20mM NaF, 20 mM β-glycerophosphate, 7.5 mM MgCl_2_, 1% Triton X-100, 2 mM EGTA, 0.1% 2-mercaptoethanol and protease inhibitors) and the C19ORF5 protein was immunodepleted by overnight incubation with 4G1 antibody adsorbed on Protein A/G+ beads. The beads were pelleted and the supernatant was subjected to a second immunodepletion as described above. For mock immunodepletion, normal mouse IgG was used instead of anti-C19ORF5 antibody. Other procedures were performed as described previously [1,7].

#### Immunofluorescent Microscopy and cell preparation

were performed as described previously [1,7]. In some cases, cells were transfected with siRNA duplexes prior to plating on coverslips. Forty-eight hours after plating, cells were fixed in 100% methanol at -20°C for 5 minutes and processed for immunostaining as described [1]. Digital images were taken under UV illumination with appropriate filter sets at 100× magnification using a LEICA DMIRE2 microscope and captured using IPLab 3.6.5a software (Scanalytics, Rockville, MD). To allow comparison of image intensity within each experiment, images for GFP-tagged NORE1A and α-tubulin were taken at the same exposure and processed identically in the Adobe Photoshop program.

#### Tubulin-Binding (microtubule cosedimentation *in vitro*) Assays

Microtubule cosedimentation assay was done with the Microtubule Binding Protein Spin Down Assay Kit (Cytoskeleton Inc. Denver, CO, cat.# BK029), according to the manufacturer's instructions. After assay, the pellet was adjusted to the same volume as the supernatant with GTB. Both supernatant and pellet were adjusted to 1 × SDS gel loading buffer with 4× stock solution. Equal volumes of supernatant and pellet were separated by SDS PAGE and analyzed by western blotting. In some experiments, purified FLAG-NORE1A was preincubated for 1 hour at 37°C with C19ORF5-immunodepleted or mock-immunodepleted HEK293 cell extract, or purified FLAG-C19ORF5 prior to incubation with microtubules.

#### Cell Cycle Distribution Assay

A549 cells were transfected with anti-MAP1B siRNA pool, or anti-C19ORF5 siRNA pool or control siRNA as descried above. After one day cells were replated in triplicate on 6-well plates and 8 hours after were infected with retrovirus expressing GFP or GFP-NORE1A effector domain (aa 191–363). Two days later, cells were fixed with 1% formaldehyde in PBS, stained with 0.05 mg/ml propidium iodide and analyzed by FACSvantage (Becton-Dickinson, San Jose, CA). Cell cycle distributions of GFP-positive cells were determined by ModFIT software.

## Abbreviations

The abbreviations are: NORE1, novel Ras effector 1; RASSF1A, Ras-association domain family 1 isoform A; HA, hemagglutinin; FLAG, peptide tag DYKDDDDK; hrGFP, *Renilla reniformis *Green Fluorescent Protein; DAPI, 4', 6-diamidino-2-phenylindole; GTB, general tubulin buffer; BSA, bovine serum albumin; kDa, kilodalton; MAP, microtubule-associated proteins.

## Competing interests

The authors declare that they have no competing interests.

## Authors' contributions

AM helped conceive of the study, carried out the experimental work, analyzed the data and participated in drafting the manuscript. SK helped conceive of the study and analyzed the data, carried out some of the experimental work, and participated in drafting the manuscript. AK conceived of the study, participated in the design of the experiments, carried out some of the experimental work, analyzed the data, wrote the first draft of the manuscript and edited it. All the authors have read and approved the final version of the manuscript.

## Supplementary Material

Additional file 1 **Supplemental Figure 1**: Distribution of tubulin in the supernatant and pellet fractions of the microtubular cosedimentation assay was examined. **Supplemental Figure 2**: Microtubular distribution of GFP-NORE1A191-363 was examined in A549 cells in which MAP1B proteins were depleted by RNA interference. **Supplemental Figure 3**: Centrosomal distribution of GFP-NORE1A191-363 was examined in A549 cells in which MAP1B proteins were depleted by RNA interference. Click here for file
