# Supplementary material for: Interaction of the growth and tumour suppressor NORE1A with microtubules is not required for its growth-suppressive function
Source: BMC Res Notes. 2008 May 15;1:13. doi: 10.1186/1756-0500-1-13 (PMC2518271; doi:10.1186/1756-0500-1-13)

Supplemental Figure 1: The distribution of tubulin in the supernatant and pellet fractions of the microtubular cosedimentation assay. Purified bovine brain tubulin (Cytoskeleton) was polymerized into microtubules in general tubulin buffer (GTB: 80 mM PIPES pH 7.1, 1 mM $MgCl_2$, 1 mM EGTA) for 20 min at 35°C. Taxol (20 µM) was added to polymerized microtubules. An extract of A549 cells expressing GFP-NORE1A was mixed with equal amounts of microtubules (lanes 1, 2) or with GTB buffer only (lanes 3, 4). Separate reactions (lanes 5, 6) contained unpolymerized tubulin to which buffer A used for cell lysis, described in Methods, was added. After incubation for 30 min at room temperature, microtubules were pelleted by centrifugation at 16,000g for 1 hour at RT and supernatant was collected. The pellet was adjusted to the same volume as the supernatant with GTB. Both supernatant and pellet were adjusted to 1xSDS gel loading buffer with 4x stock solution. Equal volumes of supernatant (S) and pellet (P) were separated by SDS PAGE and analyzed by Western blotting with anti-tubulin antibodies.

Supplemental Figure 2: Depletion of MAP1B protein interferes with microtubule localization of NORE1A and its effector domain in A549 cells. A549 cells expressing GFP-NORE1A or GFP-NORE1A 191-363 were transfected with anti-MAP1B siRNA pool or control siRNA. Cells were fixed, processed for immunofluorescence, and imaged as described in Methods. Each panel shows NORE1A, α-tubulin which was used as a microtubular marker, and a superimposed image containing an additional image of nuclei stained with DAPI. Bar, 10 µm.

Supplemental Figure 3: Depletion of MAP1B protein did not result in dissociation of NORE1A and its effector domain from centrosomes in A549 cells.

A549 cells expressing GFP-NORE1A or GFP-NORE1A 191-363 were transfected with anti-MAP1B siRNA pool or control siRNA. Cells were fixed, processed for immunofluorescence, and imaged as

described in Methods. Each panel shows NORE1A, γ-tubulin which was used as a centrosomal marker, and a superimposed image containing an additional image of nuclei stained with DAPI. Arrows indicate centrosomes. Bar, 10 μm.

Supplemental Figure 1

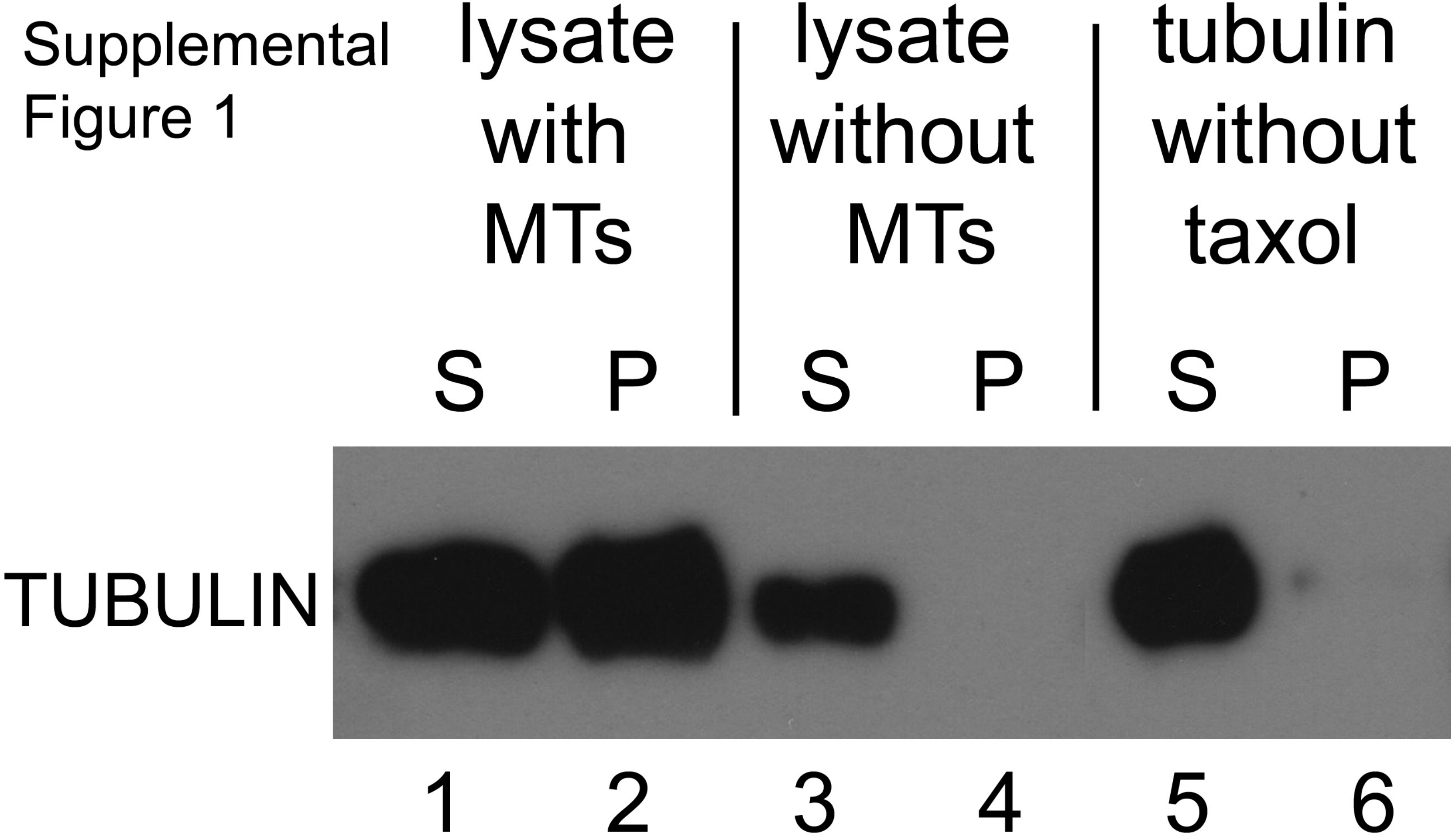

Supplemental Figure 2

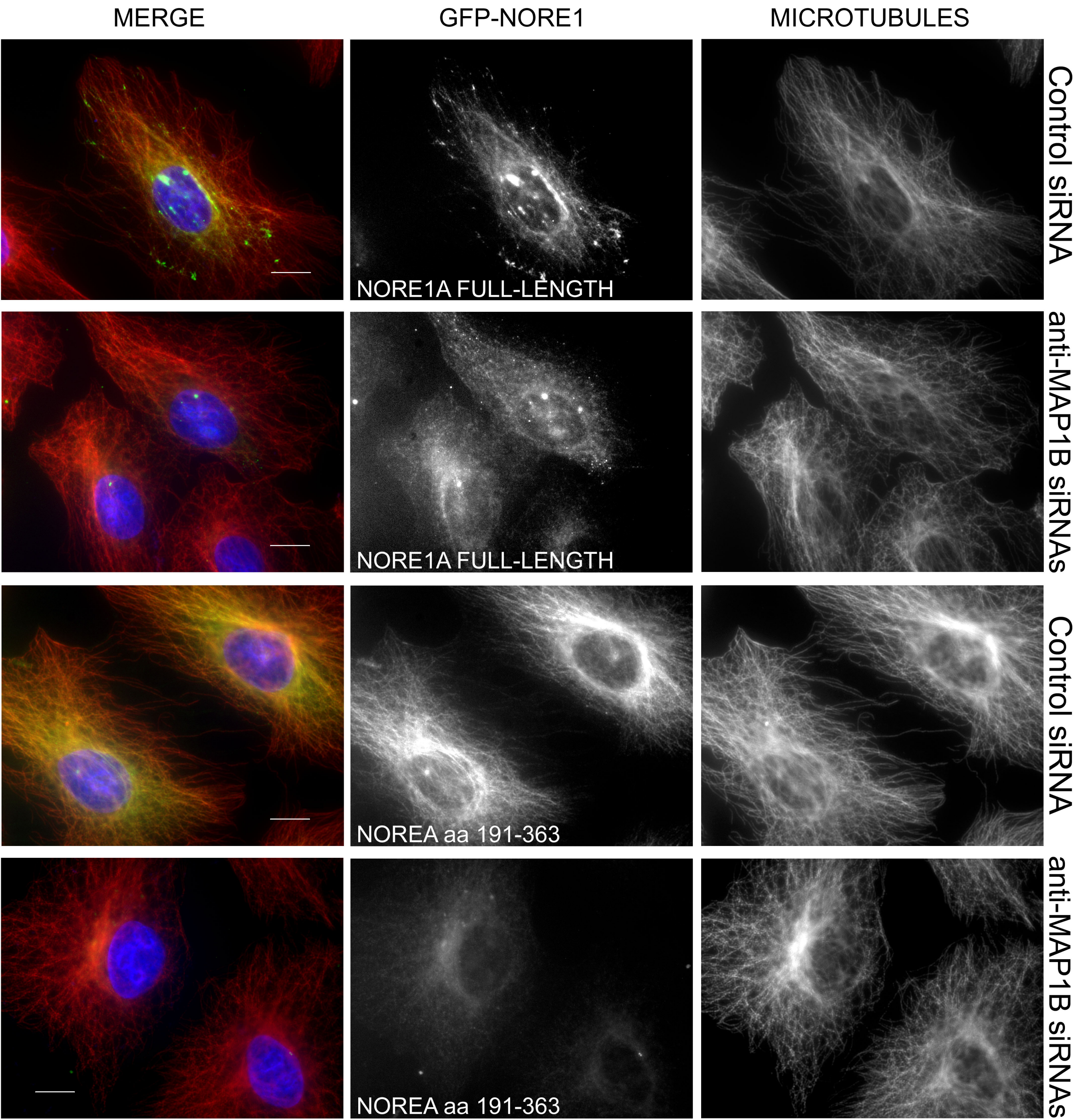

Supplemental Figure 3

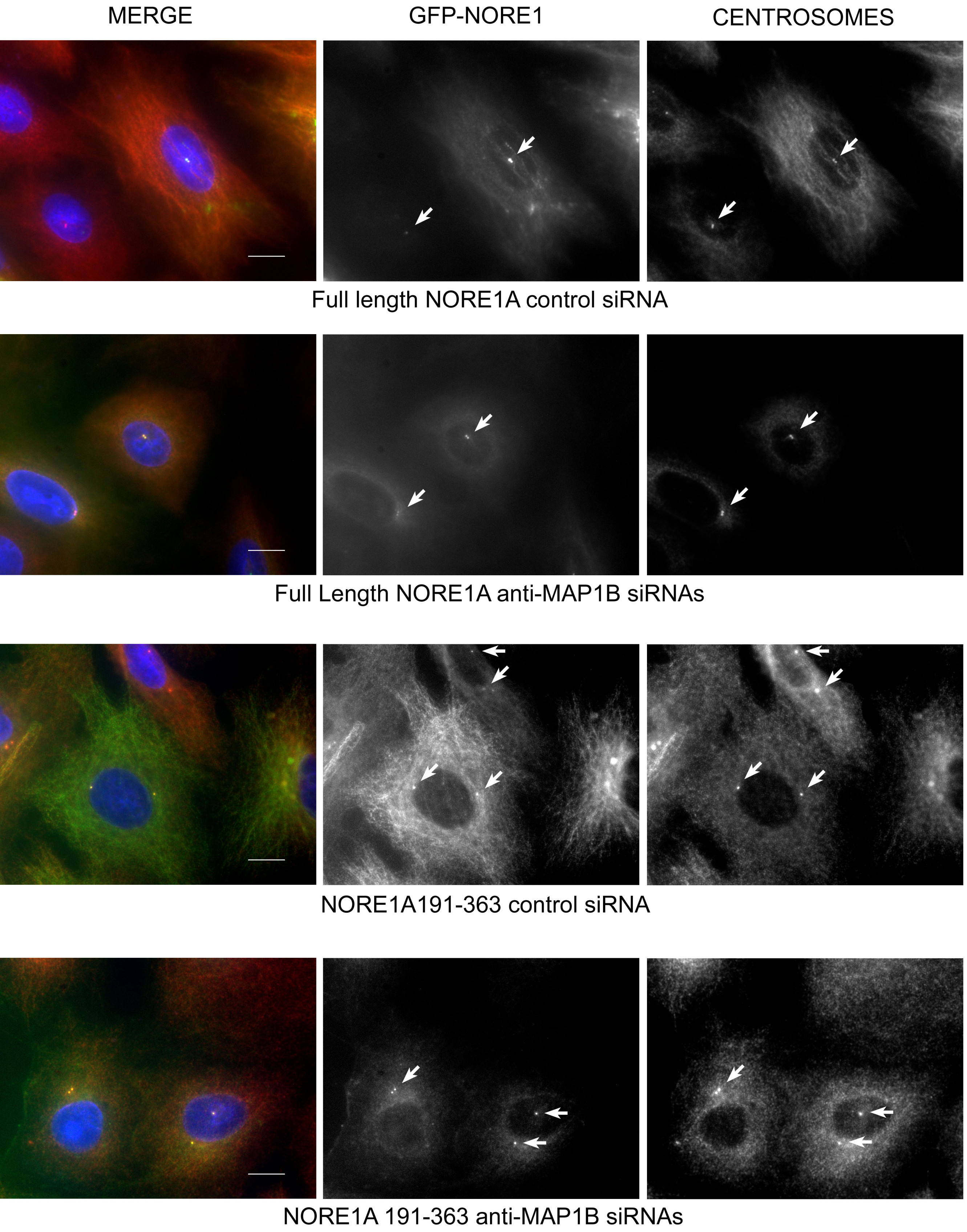

Supplement: Additional file 1 — Supplemental Figure 1: Distribution of tubulin in the supernatant and pellet fractions of the microtubular cosedimentation assay was examined. Supplemental Figure 2: Microtubular distribution of GFP-NORE1A191-363 was examined in A549 cells in which MAP1B proteins were depleted by RNA interference. Supplemental Figure 3: Centrosomal distribution of GFP-NORE1A191-363 was examined in A549 cells in which MAP1B proteins were depleted by RNA interference. [file 1756-0500-1-13-S1.pdf]
